# First International External Quality Assessment of Molecular Detection of Crimean-Congo Hemorrhagic Fever Virus

**DOI:** 10.1371/journal.pntd.0001706

**Published:** 2012-06-26

**Authors:** Camille Escadafal, Stephan Ölschläger, Tatjana Avšič-Županc, Anna Papa, Jessica Vanhomwegen, Roman Wölfel, Ali Mirazimi, Anette Teichmann, Oliver Donoso-Mantke, Matthias Niedrig

**Affiliations:** 1 Centre for Biosafety, Robert Koch Institute, Berlin, Germany; 2 European Public Health Microbiology Training Program (EUPHEM), European Centre for Disease Prevention and Control (ECDC), Solna, Sweden; 3 Bernhard-Nocht-Institut für Tropenmedizin, Hamburg, Germany; 4 Faculty of Medicine, Institute of Microbiology and Immunology, Ljubljana, Slovenia; 5 School of Medicine, Aristotle University of Thessaloniki, Thessaloniki, Greece; 6 Laboratory for Urgent Response to Biological Threats, Institut Pasteur, Paris, France; 7 Bundeswehr Institute of Microbiology, Munich, Germany; 8 Swedish Institute for Communicable Disease Control, Stockholm, Sweden; University of Leeds, United Kingdom

## Abstract

Crimean-Congo hemorrhagic fever (CCHF) is a zoonosis caused by a *Nairovirus* of the family *Bunyaviridae*. Infection is transmitted to humans mostly by *Hyalomma* ticks and also by direct contact with the blood or tissues of infected humans or viremic livestock. Clinical features usually include a rapid progression characterized by hemorrhage, myalgia and fever, with a lethality rate up to 30%. CCHF is one of the most widely distributed viral hemorrhagic fevers and has been reported in Africa, the Middle East and Asia, as well as parts of Europe. There is no approved vaccine or specific treatment against CCHF virus (CCHFV) infections. In this context, an accurate diagnosis as well as a reliable surveillance of CCHFV infections is essential. Diagnostic techniques include virus culture, serology and molecular methods, which are now increasingly used. The European Network for the Diagnostics of “Imported” Viral Diseases organized the first international external quality assessment of CCHVF molecular diagnostics in 2011 to assess the efficiency and accurateness of CCHFV molecular methods applied by expert laboratories. A proficiency test panel of 15 samples was distributed to the participants including 10 different CCHFV preparations generated from infected cell cultures, a preparation of plasmid cloned with the nucleoprotein of CCHFV, two CCHFV RNA preparations and two negative controls. Forty-four laboratories worldwide participated in the EQA study and 53 data sets were received. Twenty data sets (38%) met all criteria with optimal performance, 10 (19%) with acceptable performance, while 23 (43%) reported results showing a need for improvement. Differences in performance depended on the method used, the type of strain tested, the concentration of the sample tested and the laboratory performing the test. These results indicate that there is still a need for improving testing conditions and standardizing protocols for the molecular detection of Crimean-Congo hemorrhagic fever virus.

## Author Summary

Crimean-Congo hemorrhagic fever (CCHF) is a widely distributed viral hemorrhagic fever as the disease occurs in much of Africa, the Middle East and Asia, Balkans and Russia. CCHF is a zoonotic viral disease mainly transmitted by ticks, although animal-to-human and human-to-human transmission also occurs. CCHF is asymptomatic in animals but presents a serious threat to humans causing nonspecific flu-like symptoms that may progress to a serious hemorrhagic syndrome with a fatality rate up to 50%. There is no approved vaccine or antiviral treatment against CCHF infections and antiviral treatment relies on early confirmation of the disease. In this context, accurate diagnosis and reliable surveillance of CCHF is essential. Diagnostic techniques for CCHF virus (CCHFV) rely more and more on molecular methods, which are rapid, simple and sensitive when correctly standardized. In this study, we present the results of the first international external quality assurance (EQA) for molecular diagnosis of CCHFV. The objective of this EQA is to collect information on the efficiency and accurateness of CCHFV molecular methods applied by expert laboratories. Participants included 44 expert laboratories from 29 different countries worldwide. The study shows uneven performances indicating that there is still a need for improving testing conditions and standardizing protocols.

## Introduction

Crimean-Congo hemorrhagic fever (CCHF) is an acute and highly contagious viral disease with high case fatality rate. It is caused by CCHF virus (CCHFV), a segmented, negative-stranded RNA virus belonging to the genus *Nairovirus* in the *Bunyaviridae* family. Transmission to humans occurs mainly by tick bites (mainly of the genus *Hyalomma*), but also by exposure to body fluids or tissues of viremic patients or animals. Livestock such as cattle, sheep, goats, hares and pigs can host CCHFV without showing any symptoms [1]. In humans, CCHF typically presents with sudden onset of high fever, severe myalgia, malaise and gastrointestinal symptoms. Frequent extensive hemorrhages may occur at later stages of the disease leading to a high fatality rate (up to 50%) [2], [3].

Following dengue viruses, CCHFV is one of the most widespread medically important arboviruses. The geographic range of CCHFV includes Africa, Asia, and Eastern Europe. In Europe, CCHF is endemic in Bulgaria since the 1950s and during the last decade CCHF outbreaks and cases have increased in several Balkan countries, Ukraine, Turkey and south-western regions of the Russian Federation with significantly high fatality rates [4]–[14]. A CCHF case was notified for the first time in Greece in June 2008 [15]. Several imported cases have also been reported lately [16], [17]. Changes in climatic conditions could expand the range of its tick vectors, and increase the incidence of disease.

Several factors have made CCHFV become an important public health issue including its wide and extending geographical distribution, its potential to cause outbreaks and highly fatal disease in humans, the lack of vaccine, limited treatment options as well as fears about its use as a biological agent by terrorists or criminals.

Due to its potential to cause community and nosocomial outbreaks, a quick and accurate diagnosis of CCHF is important for case management and protection of medical staff. In fact, late diagnosis of patients decreases treatment efficacy and increases the risk of fatal outcome.

Diagnostic assays for CCHFV include virus isolation, enzyme-linked immunosorbent assays and reverse transcription–PCR (RT-PCR) [1], [3]. Virus isolation is very constraining as it must be performed in high biocontainment laboratories of biosecurity level 4 (BSL4). Molecular methods for the detection of the viral genome offer a rapid, sensitive, and highly specific alternative for early diagnosis during the viremic phase of infection or in post-mortem tissues. However, because of the remarkable genetic variability among CCHFV strains, most current RT-PCRs either fail to detect specific strains or lack sensitivity [18]. A further improvement has been the development of real-time RT-PCRs, which have higher specificity and sensitivity, higher time-effectiveness and lower risk of contamination than conventional RT-PCR [19]–[21].

The performance of the different techniques applied for molecular diagnosis of CCHFV may vary between laboratories and external quality assessment (EQA) studies to assess the quality of CCHF molecular diagnostics have not been performed until now. For this reason, the European Network for Diagnostics of ‘Imported’ Viral Diseases (ENIVD) (http://www.enivd.org) organized the first EQA study for molecular diagnosis of CCHFV in 2011 with 44 laboratories participating from 29 different countries worldwide. Such studies allow the participating laboratories to monitor the quality of current diagnosis, highlight possible weak points in particular techniques and evaluate their capacity for surveillance activities. Using the results of this study, the ENIVD can also provide support and advice to all participants and laboratories performing CCHFV molecular diagnosis.

## Materials and Methods

### Call for participation

A total of 47 laboratories involved in diagnostics of CCHF infections were invited to participate in this study. Invitees are members of the ENIVD or national/regional reference laboratories for CCHF or vector-borne diseases. The study was announced as an EQA for the molecular diagnosis of CCHFV infections involving the publication of results in a comparative and anonymous manner.

This EQA was coordinated by the ENIVD following similar procedures as during previous studies performed by the network [22]–[24].

### Specimen preparation

A proficiency test panel of 15 samples was prepared which included inactivated and stable CCHFV preparations generated from Vero E6 cell culture supernatants of 4 different CCHFV genotypes: Europe 1 (strain Hoti, isolated in Kosovo), Asia 1 (strain Afg09-2990, isolated from a human CCHFV infection acquired in Afghanistan), Africa 3 (strain ArD 39554, isolated in Mauritania) and Europe 2 (strain AP-92, isolated in Greece). Viral cell supernatants were inactivated by heating for 1 h at 60°C and gamma irradiation (25 kilogray) to assure their non-infectivity [25]–[29]. The panel also included a preparation of a plasmid (pGEM-4Z, Promega) cloned with the nucleoprotein (NP) sequence of strain CCHFV IbAr 10200 and two RNA preparations extracted from cultures of CCHFV isolates from Iran and Greece. All samples were diluted with fresh frozen plasma previously confirmed as negative for CCHFV. Aliquots of 100 µl were number-coded, freeze dried for 24 h (Christ, AlphaI-5, Hanau, Germany) and stored at 4°C until dispatch.

The EQA panel was composed of:

- 5 serial 10-fold dilutions of CCHFV positive sera, genotype Europe 1
- 3 serial 10-fold dilutions of CCHFV positive sera, genotype Asia 1
- 1 positive sample of CCHFV positive sera, genotype Europe 2
- 1 positive sample of CCHFV positive sera, genotype Africa 3
- 1 sample of CCHFV NP cloned in pGEM-4Z
- 2 samples containing extracted RNA from 2 different CCHFV isolates
- 2 negative controls

### Validation and dispatch of the panel sets

To validate the molecular panel, 3 different sets of EQA samples were tested by expert laboratories before distribution by the Robert Koch Institute (RKI), Berlin, Germany. After reconstitution with 100 µl of water, the samples were extracted using the QIAamp viral RNA minikit (Qiagen, Hilden, Germany) according to the manufacturer's instructions. As above mentioned, we estimated the CCHFV genome copies present in these samples by real time RT-PCR.

The EQA panels were distributed to participants with full instructions. Samples were shipped by normal post at ambient temperature to the participating laboratories. We requested participant laboratories to resuspend the samples in 100 µl of water and to analyze the material as serum samples for detection of CCHFV RNA. They were asked to report their results and any problems encountered as well as information on the chosen protocol using a common formulary included in the documentation.

A time-stability control of the EQA samples was additionally performed by a reference laboratory by testing the samples at two different time points with a 3 months interval.

### Evaluation of the results

To guarantee anonymous participation, an individual numerical identification code was assigned to the results reported by each laboratory. This number was followed by a letter (a, b, c, d, e) when distinguishable data sets of results based on different methods were sent.

The results were scored in reflection of sensitivity and specificity. We assigned one point for correct positive or negative result whereas false- negatives/-positives results were not scored. Equivocal or borderline results were not counted. Results for the testing of the RNA samples were not considered in the scoring system as both samples seemed to be instable and most of laboratories could not detect the presence of CCHFV RNA in these samples.

Additionally, results were classified as optimal, acceptable (only one false-negative result) or need for improvement (false-positive result and/or several false-negatives).

### Statistical analysis

Data collected were entered into Microsoft Excel (Microsoft Corp., Bellingham, WA, USA). In order to compare the performances of the different diagnostic methods, a chi-square test and simple logistic regression were performed using STATA/SE version 12.0 (StataCorp, College Station, USA). As real-time RT-PCR is the most frequently used method, it has been chosen as the reference test and its performance has then been compared to the performance of the other methods: nested RT-PCR, conventional RT-PCR, real-time combined with nested RT-PCR and real-time combined with conventional RT-PCR. Test performance was estimated by calculating the total proportion of correct result for each method. The p value <0.01 was considered statistically significant.

## Results

We obtained from the invitees a response rate of 94% representing a total of 44 participating laboratories from 29 different countries (22 European, 3 Asian and 2 American countries, 1 Eurasian and 1 African country):

Medical University of Vienna Department of Virology, Vienna, Austria; Enzootic and (re)emerging viral diseases, CODA-CERVA-VAR, Brussels, Belgium; National Reference Vector-borne infections and Leptospirosis laboratory, National Centre of Infectious and Parasitic Diseases, Sofia, Bulgaria; Special Pathogens Program National Microbiology laboratory, Winnipeg, Canada; Laboratoire P4 Jean Mérieux INSERM, Lyon, France; IRBA Virology unit, La Tronche, France; Institute of Parasitology Academy of Sciences of the Czech Republic; Bernhard Nocht Institut, Hamburg, Germany; Institut für Virologie Philipps-Universität Marburg, Germany; Diagnostic Division Bundeswehr Institute of Microbiology, Munich, Germany; Clinical University of Freiburg, Department of Virology, Freiburg, Germany; TIB MOLBIOL Syntheselabor GmbH, Berlin, Germany; Robert Koch Institut, Berlin, Germany; Institute for Novel and Emerging Infectious Diseases Friedrich-Loeffler-Institut, Greifswald, Germany; Aristotle University of Thessaloniki School of Medicine A′ Department of Microbiology, Greece; National Centre for Epidemiology virologické odd., Budapest, Hungary; Arboviruses and Viral Haemorrhagic Fevers Laboratory (National Ref. Lab) Pasteur Institute of Iran; Central Virology Laboratory Ministry of Health, Public Health Laboratories Sheba Medical Centre, Israel; Laboratory CRREM - U.O. Microbiologia - Policlinico S. Orsola-Malpighi, Bologna, Italy; Padiglione Baglivi National Institute for Infectious Diseases “L. Spallanzani”, Rome, Italy; Istituto Zooprofilattico Sperimentale dell'Abruzzo e del Molise “G. Caporale”, Teramo, Italy; Viral Zoonoses Unit Dept. Veterinary public health & Food safety, Istituto Superiore di Sanità, Rome, Italy; Infectiology Centre of Latvia, Riga, Latvia; Centre for Vectors and Infectious Diseases Research National Institute of Health, Aguas de Moura, Portugal; Arbovirus and VHF Diagnostic Activity Laboratory of Vector-Borne Infections Cantacuzino Institute, Bucharest, Romania; Central Research Institute of Epidemiology, Moscow, Russia; King Fahd Medical Research Centre; King Abdulaziz University, Jeddah, Saudi Arabia; University of Ljubljana, Faculty of Medicine, Institute of Microbiology and Immunology, Ljubljana, Slovenia; Arbovirus and Imported Viral Disease Unit Centro Nacional de Microbiologia Instituto de Salud Carlos III, Majadahonda, Spain; Area de Enfermedades Infecciosas, Hospital San Pedro, Logrono, Spain; Departamento de Microbiología, Hospital Clinic y Provincial de Barcelona, Spain; Laboratorio Central de Veterinaria de Algete, Madrid, Spain; Special Pathogens Unit National Institute for Communicable Diseases of the National Health Laboratory Service, Johannesburg, South Africa; Swedish Institute for Communicable Disease Control, Solna, Sweden; Laboratory of Virology University of Geneva Hospitals, Geneva, Switzerland; Virology, Spiez laboratory, Switzerland; Erasmus MC Dept. Virology, Rotterdam, The Netherlands; Erzurum Hıfzıssıhha Enstitüsü Müdürlüğü, Turkey; Samsun Regional Hygiene Centre, Turkey; Virology Departement. Refik Saydam National Public Health Agency, Ankara, Turkey; Firat University, Veterinary Medicine, Department of Virology, Elazig, Turkey; Special Pathogens Reference Unit Microbiology Services Health Protection Agency, Salisbury, United Kingdom; Laboratory of Virology, Rocky Mountain Laboratories, Hamilton, United States of America; Viral Special Pathogens Branch, Infectious Diseases, CDC, Atlanta, United States of America.

Most of these laboratories cannot provide the BSL4 conditions required to handle infectious CCHFV. Nevertheless they have all the safety requirements to perform molecular detection of CCHFV and more specifically this EQA as all testing material provided is non-infectious.

A total of 53 data sets were received including 3 double sets from laboratories using 2 methods (sets 13a/b, 34a/b, 35a/b), 1 triple set (2a/b/c) and 1 quintuple data set (42a/b/c/d/e).

A variety of tests were used for detection and identification of CCHFV genome by participating laboratories. Among the 53 datasets received, we recorded the use of real-time RT- PCR (n = 36, 68%), RT-nested PCR (n = 5, 9%), conventional RT-PCR (n = 2, 4%), real-time RT-PCR combined with nested RT-PCR (n = 4, 8%) and real-time RT-PCR combined with conventional RT-PCR (n = 6, 11%) (Table 1 and 2).

**Table 1:** Results of the EQA for molecular detection of CCHFV – Part 1.

origin	Kosovo Hoti	Kosovo Hoti	Kosovo Hoti	Kosovo Hoti	Kosovo Hoti	Afg 09-2990	Afg 09-2990	Afg 09-2990	ArD 39554	AP-92	pGEM-4Z	neg.	neg.			
dilution	1∶10E2	1∶10E3	1∶10E4	1∶10E5	1∶10E6	1∶10E2	1∶10E3	1∶10E4	1∶10E2	1∶10E2	1∶10E4	none	none			
lab n°	#2	#9	#12	#4	#14	#10	#5	#13	#1	#6	#17	#7	#8	score	classification	method
**2b**	+*	+*	+*	+*	+*	+*	+*	+*	+*	+*	+*	−	−	13	Optimal	nRT (35)
**29**	**+** *	**+** *	**+** *	**+** *	**+** *	**+** *	**+** *	**+** *	**+** *	**+** *	**+** *	−	−	13	Optimal	cRT+qRT
**42a**	**+** *	**+** *	**+** *	**+** *	**+** *	**+** *	**+** *	**+** *	**+** *	**+** *	**+** *	−	−	13	Optimal	qRT (17)
**33**	**+** *	**+** *	**+** *	**+** *	**+** *	**+** *	**+** *	+	**+** *	**+** *	**+** *	−	−	13	Optimal	nRT(31)+qRT(30)
**31**	**+** *	**+** *	**+** *	**+** *	**+** *	**+** *	+	+	+	+*	+*	−	−	13	Optimal	qRT(32)
**11**	**+** *	**+** *	**+** *	**+** *	**+** *	+	+	+	+	**+** *	+	−	−	13	Optimal	qRT(30+32+h)
**3**	+	+	+	+	+	+	+	+	+	+	+	−	−	13	Optimal	qRT
**13a**	+	+	+	+	+	+	+	+	+	+	+	−	−	13	Optimal	nRT
**13b**	+	+	+	+	+	+	+	+	+	+	+	−	−	13	Optimal	qRT (30)
**16**	+	+	+	+	+	+	+	+	+	+	+	−	−	13	Optimal	qRT (30)
**17**	+	+	+	+	+	+	+	+	+	+	+	−	−	13	Optimal	qRT
**18**	+	+	+	+	+	+	+	+	+	+	+	−	−	13	Optimal	cRT+qRT(30)
**19**	+	+	+	+	+	+	+	+	+	+	+	−	−	13	Optimal	qRT (c)
**22**	**+**	**+**	**+**	**+**	**+**	**+**	**+**	**+**	**+**	**+**	**+**	−	−	13	Optimal	qRT (30)
**23**	**+**	**+**	**+**	**+**	**+**	**+**	**+**	**+**	**+**	**+**	**+**	−	−	13	Optimal	qRT (30)
**27**	+	+	+	+	+	+	+	+	+	+	+	−	−	13	Optimal	cRT+qRT
**35a**	+	+	+	+	+	+	+	+	+	+	+	−	−	13	Optimal	qRT (c)
**35b**	+	+	+	+	+	+	+	+	+	+	+	−	−	13	Optimal	qRT (c)
**36**	+	+	+	+	+	+	+	+	+	+	+	−	−	13	Optimal	nRT(35)+qRT
**43**	+	+	+	+	+	+	+	+	+	+	+	−	−	13	Optimal	qRT
**28**	**+** *	**+** *	**+** *	**+** *	**+** *	**+** *	**+** *	FN	**+** *	**+** *	**+** *	−	−	12	Acceptable	qRT
**2c**	+	+	+	+	+	+	+	FN	+	+	+	−	−	12	Acceptable	qRT (c)
**4**	+	+	+	+	+	+	+	FN	+	+	+	−	−	12	Acceptable	qRT
**9**	+	+	+	+	+	+	+	FN	+	+	+	−	−	12	Acceptable	qRT
**21**	+	+	+	+	+	+	+	FN	+	+	+	−	−	12	Acceptable	qRT (30)
**25**	+	+	+	+	+	+	+	FN	+	+	+	−	−	12	Acceptable	qRT (c)
**26**	+	+	+	+	+	+	+	FN	+	+	+	−	−	12	Acceptable	qRT

+: positive (17): Midilli et al., 2007.

−: negative (30): Wölfel et al., 2007.

**bold**: quantified result (32): Drosten et al., 2002.

*: : correct strain (35): Rodriguez et al., 1997.

FN: false negative result (36): Schwarz et al., 1996.

qRT: real-time RT-PCR (c): commercial assay.

nRT: nested RT-PCR (h): in house assay.

cRT: conventional RT-PCR.

**Table 2:** Results of the EQA for molecular detection of CCHFV – Part 2.

origin	Kosovo Hoti	Kosovo Hoti	Kosovo Hoti	Kosovo Hoti	Kosovo Hoti	Afg 09-2990	Afg 09-2990	Afg 09-2990	ArD 39554	AP-92	pGEM-4Z	neg.	neg.			
dilution	1∶10E2	1∶10E3	1∶10E4	1∶10E5	1∶10E6	1∶10E2	1∶10E3	1∶10E4	1∶10E2	1∶10E2	1∶10E4	none	none			
lab n°	#2	#9	#12	#4	#14	#10	#5	#13	#1	#6	#17	#7	#8	score	classification	method
**37**	+	+	+	+	+	+	+	FN	+	+	+	−	−	12	Acceptable	qRT (c)
**39**	+	FN	+	+	+	+	+	+	FN	+	+	−	−	12	Acceptable	qRT (30)
**1**	+	+	+	+	+	+	FN	+	+	+	+	−	−	12	Acceptable	qRT
**32**	+	+	+	+	+	+	+	+	+	+	+	FP	−	12	Improve	qRT (30)
**6**	+	+	+	+	+	++	+	+	FN	+	++	FP	−	11	Improve	nRT(36)+qRT(34)
**8**	+	+	+	+	+	+	+	+	FN	FN	+	−	−	11	Improve	qRT (c)
**10**	+	+	+	+	+	+	+	+	FN	+	+	FP		11	Improve	cRT+qRT
**34a**	+	+	+	+	+	+	+	FN	+	+	+	−	FP	11	Improve	cRT+qRT
**44**	+	+	+	+	+	+	+	+	+	FN	+	FP	−	11	Improve	qRT
**14**	+	+	+	+	+	+	+	FN	+	FN	FN	−	−	10	Improve	qRT (h)
**30**	+	+	+	+	+	+	FN	FN	FN	+	+	−	−	10	Improve	qRT (c)
**38**	+	+	FN	FN	FN	+	+	+	+	+	+	−	−	10	Improve	qRT (30)
**42b**	+	+	+	+	FN	+	FN	FN	+	+	+	−	−	10	Improve	nRT (33a)
**7**	+	**+** *	**+** *	**+** *	**+** *	FN	FN	FN	FN	+	+	−	−	9	Improve	cRT(38)+qRT(21)
**20**	+	+	+	+	FN	+	+	+	+	FN	+	FP	FP	9	Improve	nRT+qRT
**24**	+	+	+	+	+	FN	FN	FN	FN	+	+	−	−	9	Improve	qRT
**2a**	**+**	**+**	**+**	**+**	**+**	FN	FN	FN	FN	FN	FN	−	−	7	Improve	qRT (35)
**5**	+	+	+	+	+	FN	−	FN	FN	FN	FN	−	−	7	Improve	qRT (34)
**12**	+	+	+	FN	FN	+	FN	FN	FN	FN	+	−	−	7	Improve	qRT (32)
**42d**	+	+	FN	FN	FN	+	FN	FN	FN	+	+	−	−	7	Improve	cRT (39)
**34b**	FN	FN	FN	FN	FN	+	+	FN	+	FN	+	−	−	6	Improve	qRT
**15**	FN	FN	FN	FN	FN	+	FN	FN	FN	+	+	−	−	5	Improve	qRT (30)
**40**	+	+	FN	FN	FN	FN	FN	FN	FN	FN	+	−	−	5	Improve	cRT
**41**	FN	+	+	FN	FN	FN	FN	FN	+	FN	FN	−	FP	4	Improve	qRT
**42c**	FN	FN	FN	FN	FN	FN	FN	FN	FN	+	FN	−	−	3	Improve	nRT (33b)
**42e**	FN	FN	FN	FN	FN	FN	FN	FN	FN	FN	FN	−	−	2	Improve	nRT (37)
**% correct**	**91**	**91**	**87**	**83**	**79**	**85**	**75**	**55**	**77**	**81**	**89**	**89**	**94**			

+: positive (21): Duh et al., 2006.

−: negative (30): Wölfel et al., 2007.

n.d.: not determined (32): Drosten et al., 2002.

**bold**: quantified result (33a): Midilli et al., 2009 (Europe 1).

*: : correct strain (33b): Midilli et al., 2009 (Europe 2).

FN: false negative result (34): Papa. et al., 2007.

PN: false positive result (35): Rodriguez et al., 1997.

qRT: real-time RT-PCR (36): Schwarz et al., 1996.

nRT: nested RT-PCR (37): Deyde et al., 2006.

cRT: conventional RT-PCR (38): Burt et al.,1998.

(c): commercial assay (39): Burt et al., 2005.

(h): in house assay.

Performance varied among laboratories and scores ranged from 2 to the maximum value of 13. Optimal results were reported by 38% (n = 20) of the analyses; 19% (n = 10) of the analyses achieved acceptable results due to the inability to detect one positive sample, and 43% (n = 23) revealed several false negative and/or one or more false positive results showing that there is room for improvement (Table 1 and 2).

Only 15% of the methods could detect the presence of CCHFV RNA in the RNA dilutions (samples #3 and #11). These results as well as previous experiences, indicate us that such RNA preparations are instable and unsuited to EQA studies. For this reason these samples have not been included in the rest of the evaluation and scoring system (data not shown).

Statistical analysis shows us that test performance is significantly associated to the method used, as the p-value is <0.0001 (Table 3). The confidence intervals indicate that conventional RT-PCR and nested RT-PCR are significantly worst-performing than real-time RT-PCR (OR<1 and 1 is not included in the confidence interval). Real-time combined with nested RT-PCR and real-time combined with conventional RT-PCR are not significantly performing differently that real-time RT-PCR only (OR>1 but 1 is not included in the confidence interval).

**Table 3:** Odd ratios obtained for each method by using real-time PCR as the reference.

Method	Proportion of correct results		OR (95% CI)	*P-*value
	No	%		
Real-time RT-PCR	398/468	85,0%	1.00 (reference)	<0.0001
Conventional RT-PCR	43/65	66,2%	0.34 (0.19–0.61)	
Nested RT-PCR	12/26	46,2%	0.15 (0.07–0.34)	
Real-time+conventional RT-PCR	46/52	88,5%	1.35 (0.55–3.28)	
Real-time+nested RT-PCR	70/78	89,7%	1.54 (0.71–3.34)	

OR: odd ratio; CI: confidence interval.

Information on the type of protocol used was requested to the participants and 58% of the data sets included this information. For the real-time PCR methods, 12 laboratories have reported to use the protocol from Wölfel et al., 2007 [30] or 2009 [31]; 3 used the protocol from Drosten et al., 2002 [32]; 3 used the protocol from Midilli et al., 2007 [17] and 2009 [33]; 2 used the protocol from Papa. et al., 2007 [34] and 2 used the protocol from Duh et al., 2006 [21]. Real-time PCRs were also performed with 3 commercial kits: 4 data sets reported the use of the Real Star CCHFV RT-PCR kit (1.0 and 1.2) from Altona diagnostics, 2 used the CCHFV real time RT-PCR kit from Shangai ZJ Bio-Tech Co. and 1 used the LightMix Kit Crimean-Congo Virus of TIB MOLBIOL. For the nested PCR methods, 2 laboratories have reported to use the protocol from Rodriguez et al., 1997 [35]; 2 used the protocol from Schwarz et al., 1996 [36]; 1 used the protocol from Deyde et al., 2006 [37]. For the one-step PCR methods, 1 laboratory has reported to use the protocol from Burt et al., 1998 [38]; 1 used the protocol from Burt et al., 2005 [39]. The methods employed for each analyze are specified in Table 1 with the reference number.

Laboratories using the same technique differed in their performance, indicating differences in individual operational procedures rather than limitations of the method itself. This is the case of the 12 laboratories which used the real-time PCR developed by Wölfel et al [30], where the corresponding scores ranged from 5 to 13. On the other hand, low performances are observed for particular methods, thus indicating the need for up-dating or improving the technique. This can be observed for the one step PCR from Burt et al [39] and the real-time PCR from Duh et al [21]. In fact these protocols do not seem to be able to detect the Afg 09-2990 and ArD 39554 strains. Although this information should be interpreted with precaution as the number of laboratories reporting the use of these methods is low (n = 2).

The sensitivity of the different diagnostic methods might be assessed by comparing the testing results of the serial dilutions of CCHFV-Hoti (samples #2, #9, #12, #4, and #14) and CCHFV Afg09-2990 (samples #10, #5 and #13). Looking at the percentage of correct results for each sample (Table 1 and 2), we observe a clear correlation between increased dilution of the sample and low sensitivity in RNA detection. This decreased sensitivity is the main reason for reporting false negatives. In fact, considering test specificity, we observe no major differences in performance when comparing the testing results obtained for the different strains and genotypes of CCHFV. However, some results indicate that specificity issues might be encountered by rarely used methods such as the protocol from Burt et al [39] and from Duh et al [21] mentioned in the previous paragraph.

We can also have indications on the specificity of the diagnostic methods looking at the testing results of the two negative controls (samples #7 and #8). As much as 13% of the data sets (7 out of 53) reported one or two false positives. All of the corresponding methods involved real-time PCR but using different protocols.

In order to estimate experience in viral load determination, further information was requested to the participants on the number of copies of CCHFV genome detected in the samples. Over half of the data sets (27 out of 53) reported quantitative results (data not shown). Among these, 13 reports gave their results as Ct values proving very limited data to estimate accurately viral load in the absence of calibrated standards. Interestingly, as much as 41% of the analyses which mentioned the use of real time-based procedures (19 out of 46) did not provide information on the viral load although the use of this method could provide such quantitative (or at least semi-quantitative) results.

In order to estimate experience in sequencing and strain typing, strain specification of the CCHFV detected in each sample was requested to the participants. Only 15% (8 out of 53) of the data sets reported completely or partially this information (data not shown). Providing only partial data was mostly due to the inability to type the Afg 09-2990 and the ArD 39554 strains. In fact out of 8 laboratories performing sequencing, 3 could not type the ArD 39554 strain and 4 could not type the Afg 09-2990 strain in all dilutions.

The stability of EQA samples was assessed by testing the samples upon reception in July 2011 and then 3 months later after storage at room temperature. Excluding the negative results of the extracted RNA samples, the results indicate that all samples were stable for a 4 month period (data not shown) and thus demonstrated that failure in CCHFV RNA detection experienced by some of the laboratories was probably not due to genome degradation during shipment or storage.

## Discussion

From this study, we can conclude that RNA preparations are not suitable for such EQA studies. In contrast, the plasmid preparation seems to be a very useful tool for the evaluation of diagnostic performance. Furthermore, plasmid preparation does not require manipulating infectious material which is a great advantage specially when handling deadly agents such as CCHFV.

From the results of this EQA, it appears clearly that the type of method used was not the main factor affecting the quality of the test results. It seems that performance is mostly linked to the reporting laboratories and their use of the different protocols since their performance differ greatly even when using the same technique. This result emphasizes the need to revise the protocols and procedures performed in laboratories with unsatisfactory results. Moreover, the study cannot designate precisely the best methods since several factors including primers, enzymes, buffers, thermocyclers, reaction conditions and genome target all have an influence on test performance. The influence of this variability is difficult to estimate but can be minimized by standardizing protocols including controls and optimizing testing conditions.

However, statistical analysis indicates that real-time RT-PCR is significantly best-performing than conventional RT-PCR and nested RT-PCR. Real-time PCR is also the most time effective method and the only method which enables to quantify directly the number of genome copies in each sample. However, the major limitation for the implementation of these assays in endemic areas is the cost of thermocyclers and reagents which hinder generalized application in the field. The statistical analysis also enabled us to conclude that combining real-time RT-PCR with another RT-PCR method is not showing any significant advantage compared to the use of real-time RT-PCR only.

CCHF has a low incidence but the disease can be very severe and cause death. Therefore, the sensitivity of diagnostic tests is of utmost importance. Reporting false negatives should be considered more critical than reporting false positives as positive results are few and can always be submitted to further testing for confirmation. Regarding false positive results using real time PCR it is critical to evaluate properly the obtained curve and the Ct value, and in doubtful occasions to proceed in gel electrophoresis of the PCR products, as these false positive results are rather due to non-specific product rather than to cross contamination.

Most of the participants provided no information regarding strain typing. However correct results without strain specification are completely satisfactory in the context of clinical diagnosis as there is no specific treatment based on each CCHFV strain. On the other hand, information on the strain type is relevant for surveillance activities in order to monitor which strain types are circulating in CCHFV-endemic areas and which type of illness is associated with these strains.

Information on the quantity of CCHFV RNA in human plasma samples can be very useful for pathogenesis studies and for monitoring the progress of clinical manifestations. In fact, it has been shown in several studies that viral load is a useful predictor of disease progress, with high viral load tending to indicate fatal outcome [30], [34], [40], [41]. In our study, most data sets (87%) reported the use of real-time PCR which enables at least semi quantitative RNA quantification. However, less than 60% of these results provided this information as RNA concentrations or as cut-off values, indicating there is still room for improvement concerning viral load determination as mentioned in previous EQA studies [23].

The increasing importance of this disease in Africa, Asia and the Middle-East, and the risk of expansion to other areas such as Europe makes necessary to assure that the methods used for the diagnosis and surveillance of CCHFV are working properly where they are already implemented. Indeed the low participation from endemic countries in this EQA, even if thoroughly announced, is pointing out the need to encourage more laboratories to implement CCHFV diagnosis techniques suited to their capacity and capability and to participate routinely in quality assurance programs.

In order to reach optimal performances for CCHFV molecular diagnosis in reference laboratories, we recommend performing such EQA studies on a regular basis. Future EQA studies should include a range of CCHFV genotypes and concentrations to reflect more accurately diagnostic performances in expert laboratories. Additionally, the EQA instructions should clearly mention to participants that complete information on the methods used is required in order to obtain better results and accurate conclusions.
